# Hierarchical Pooling Strategy Optimization for Accelerating Asymptomatic COVID-19 Screening

**DOI:** 10.1109/OJCS.2020.3036581

**Published:** 2020-11-06

**Authors:** Keqin Li

**Affiliations:** ^1^ College of Information Science and EngineeringHunan University 12569 Changsha 410082 China; ^2^ Department of Computer ScienceState University of New York 14821 New Paltz NY 12561 USA

**Keywords:** Asymptomatic screening, COVID-19, group test, hierarchical pooling strategy optimization, sample pooling, speedup

## Abstract

Testing has been a major factor that limits our response to the COVID-19 pandemic. The method of sample pooling and group test has recently been introduced and adopted. However, it is still not clearly known how to determine the appropriate group size. In this paper, we treat asymptomatic COVID-19 screening acceleration as an optimization problem, and solve the problem using an analytical approach and an algorithmic procedure. We develop a two-level hierarchical pooling strategy for accelerating asymptomatic COVID-19 screening. In the first level, a population is divided into groups, which results in inter-group acceleration. In the second level, a group is divided into subgroups, which results in intra-group and inter-subgroup acceleration. By using our analytical methods and numerical algorithms, we determine the optimal group size and the optimal subgroup size, which minimize the total number of tests, maximize the speedup of the hierarchical pooling strategy, and minimize both time and cost of testing. It is discovered that the optimal group size and the optimal subgroup size are determined by the fraction of infected people. Furthermore, the optimal group size, the optimal subgroup size, and the achieved speedup grow sublinearly with the reciprocal of the fraction of infected people. Our research has important social implications and financial impacts. For example, if the fraction of infected people is 0.01, by using group size of 25 and subgroup size of 5, we can achieve speedup of at least 11, which means that months of testing time can be reduced to days, and over 91% of the testing cost can be saved. Such results have not been available in the known literature. The paper makes significant progress and great advance in pooling strategy optimization for accelerating asymptomatic COVID-19 screening, and represents the contribution of computer science to the global pandemic.

## I. Introduction

### A. Background and Motivation

A coronavirus test requires a number of time consuming steps in the laboratory, which can take several hours. Testing has been a major factor that limits our response to the COVID-19 pandemic [1]. As governments reopen more businesses and public spaces, the number of infected people will surge, especially when there are asymptomatic people [2].

The method of sample pooling and group test has recently been introduced [7], [8] and adopted [4], [5]. The strategy involves pooling samples from multiple people. If the test result of a group of $k$ ($k\geq 2$) samples is negative, we know that all the individual samples are negative. If the test result of a group of samples is positive, then the individual samples need to be tested one by one. If the percentage of infected people is low, this pooling method can potentially significantly reduce the required number of tests and substantially save the necessary cost of tests. For example, recently, the City of Wuhan successfully screened 300 asymptomatic individuals from 9,899,828 people in only 19 days (May 14 – June 1, 2020), by using the pooling method with group size of five, involving 63 testing laboratories, 1,451 scientists and professionals, and 701 examination equipments (24 hours a day without interruption), and reaching a peak testing capacity of 1 million per day^1^^1^http://www.xinhuanet.com/local/2020-06/03/c_1126066386.htm..

However, it is still not clearly known how to determine the appropriate group size, although some attempt has been made. For instance, it has been recommended that the batch size should be powers of two [9], which depends on the frequency of positive samples out of all samples. It is clear that the choice of the best group size can reduce the time and cost of testing to the maximum extent, and therefore, will have tremendous practical impact to COVID-19 detection, prevention, response, and control.

### B. New Contributions

In this paper, we treat asymptomatic COVID-19 screening acceleration as an optimization problem, and solve the problem using an analytical approach and an algorithmic procedure. The main contributions of the paper can be summarized as follows.

• We develop a *two-level hierarchical pooling strategy* for accelerating asymptomatic COVID-19 screening. In the first level, a population is divided into groups, which results in inter-group acceleration. In the second level, a group is divided into subgroups, which results in intra-group and inter-subgroup acceleration.
• By using our analytical methods and numerical algorithms, we determine the optimal group size and the optimal subgroup size, which minimize the total number of tests, maximize the speedup of the hierarchical pooling strategy, and minimize both time and cost of testing.
• It is discovered that the optimal group size and the optimal subgroup size are determined by the fraction of infected people. Furthermore, the optimal group size, the optimal subgroup size, and the achieved speedup grow sublinearly with the reciprocal of the fraction of infected people.
• Our research has important social implications and financial impacts. For example, if the fraction of infected people is 0.01, by using group size of 25 and subgroup size of 5, we can achieve speedup of at least 11, which means that months of testing time can be reduced to days, and over 91% of the testing cost can be saved.

Such results have not been available in the known literature. The paper makes significant progress and great advance in pooling strategy optimization for accelerating asymptomatic COVID-19 screening, and represents the contribution of computer science to the global pandemic. (Note: This paper is a substantially extended version of an earlier work reported in [6], where only a one-level pooling strategy was studied.)

In Section II, we consider inter-group (i.e., group level) acceleration, and find the optimal group size. In Section III, we consider intra-group (i.e., subgroup level or inter-subgroup) acceleration, and find the optimal subgroup size for a given group size. In Section IV, we conduct joint optimization for both inter-group acceleration and intra-group acceleration, and simultaneously find the optimal group size and the optimal subgroup size. In Section V, we address some practical issues. In Section VI, we conclude the paper.

## II. Inter-Group Acceleration

In this section, we develop our method to find the optimal group size by using *inter-group acceleration*.

### A. The Method

We define the following quantities.

• $p_0$: the probability that one individual test result is positive;
• $q_0$: the probability that one individual test result is negative;
• $p_1$: the probability that one group test result is positive;
• $q_1$: the probability that one group test result is negative.

The value of $p_0$ is given and known in advance. It is clear that $q_0=1-p_0$. Furthermore, we have $q_1=q_0^k=(1-p_0)^k$, and $p_1=1-q_1=1-q_0^k=1-(1-p_0)^k$.

For a single group of samples, if the test result of the group is negative (which happens with probability $q_1$), only one test is enough; if the test result of the group is positive (which happens with probability $p_1$), $(k+1)$ tests are required, one for group test, and $k$ for individual tests. Hence, the expected number of tests for one group using the pooling method is

\begin{eqnarray*}
T_{\text{group}} &=&1\times q_1+(k+1)\times p_1\\
&=&q_0^k+(k+1)(1-q_0^k)\\
&=&k+1-kq_0^k.
\end{eqnarray*}

The total number of tests for a population of $N$ (which is divided into $N/k$ groups and $N\gg k$) is

\begin{eqnarray*}
T_{\text{pooling}} &=&\left({N\over k}\right)T_{\text{group}}\\
&=&\left({N\over k}\right)(k+1-kq_0^k)\\
&=&N\left(1+{1\over k}-q_0^k\right).
\end{eqnarray*}

Since the number of tests without using pooling is $N$, the *speedup* of the pooling strategy is

\begin{eqnarray*}
S(k) &=&{N\over T_{\text{pooling}}}\\
&=&{k\over T_{\text{group}}}\\
&=&{1\over \displaystyle {1+{1\over k}-q_0^k}}.
\end{eqnarray*}

Our objective is to maximize the speedup.

It is clear that maximizing $S(k)$ is equivalent to minimizing

\begin{equation*}
F(k)={1\over k}-q_0^k.
\end{equation*}

Note that

\begin{equation*}
{\partial F(k)\over \partial k}=-{1\over k^2}-q_0^k\ln q_0.
\end{equation*}

To have ${\partial F(k)/\partial k}=0$, we need

\begin{equation*}
{1\over k^2}=q_0^k\ln {1\over q_0},
\end{equation*}

which implies that $k$ satisfies

\begin{equation*}
k=\sqrt{\displaystyle {\left({1\over q_0}\right)^k}\over \displaystyle {\ln {1\over q_0}}}.
\end{equation*}

Unfortunately, it is not clearly known how to find an analytical and closed-form solution to the above equation of $k$ at this stage.

We now develop a numerical algorithm to find $k$. We define

\begin{equation*}
G(k)=k-\sqrt{1\over q_0^k\ln (1/q_0)}=k-{1\over \sqrt{\ln (1/q_0)}}\left({1\over \sqrt{q_0}}\right)^k.
\end{equation*}

Our purpose is to solve the equation $G(k)=0$. Noticed that

\begin{equation*}
{\partial G(k)\over \partial k}=1-{1\over \sqrt{\ln (1/q_0)}}\left(\ln {1\over \sqrt{q_0}}\right)\left({1\over \sqrt{q_0}}\right)^k,
\end{equation*}

and

**Fig. 1.**
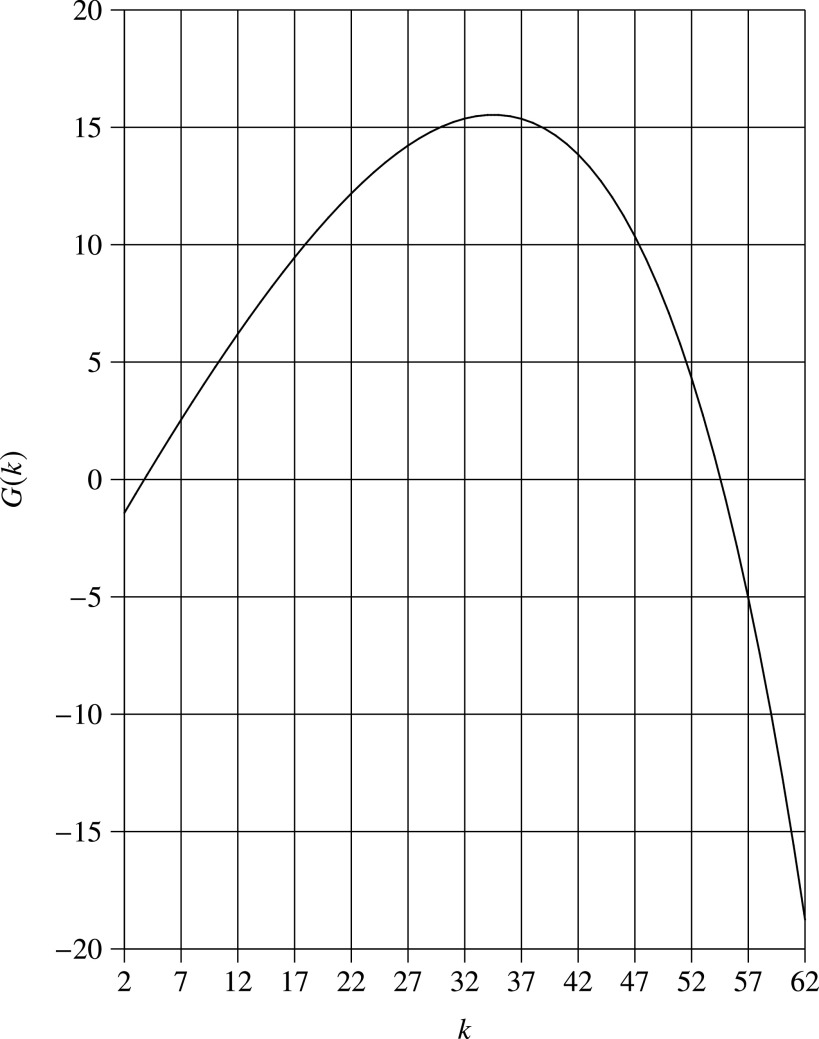
$G(k)$ vs. group size ($p_0=0.1$).

\begin{equation*}
{\partial ^2 G(k)\over \partial k^2}=-{1\over \sqrt{\ln (1/q_0)}}\left(\ln {1\over \sqrt{q_0}}\right)^2\left({1\over \sqrt{q_0}}\right)^k< 0,
\end{equation*}

which implies that $G(k)$ is a concave function. Figure 1 illustrates $G(k)$ for $p_0=0.1$. It is observed that $G(k)$ is an increasing function of $k$ when $k$ is less than 35, and $G(k)$ is a decreasing function of $k$ when $k$ is greater than 35. In other words, there are two solutions to the equation $G(k)=0$. One is between 3 and 4, and the other is between 54 and 55.

**Fig. 2.**
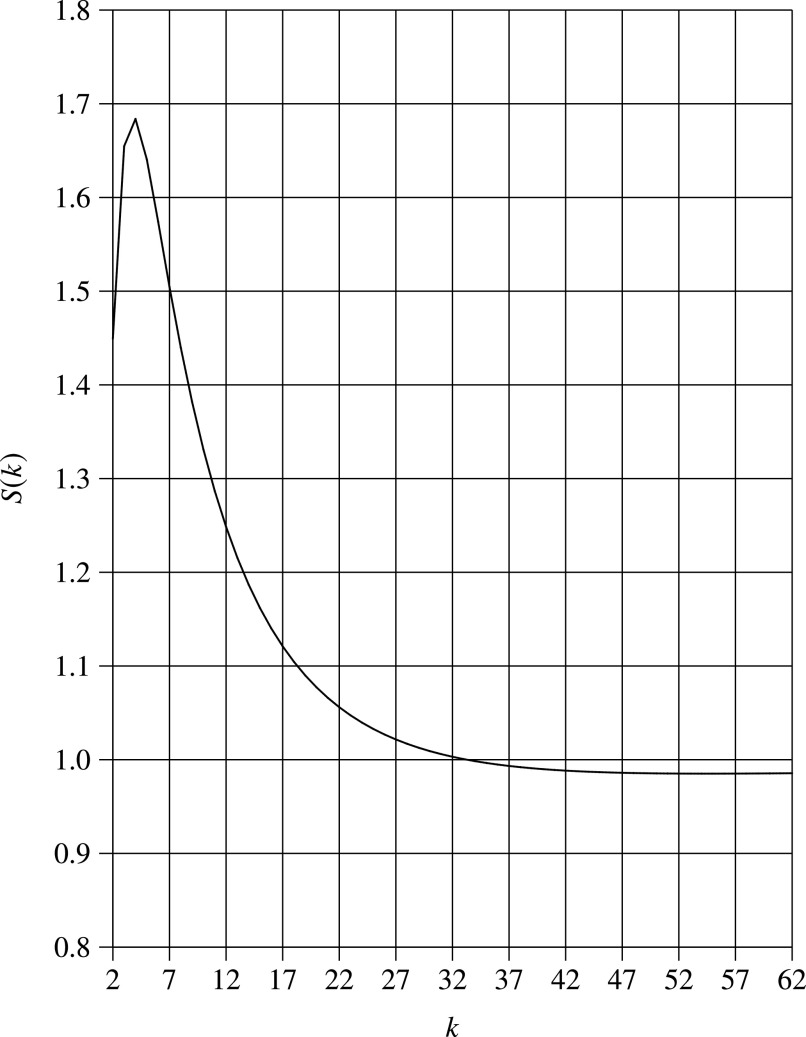
$S(k)$ vs. group size ($p_0=0.1$).

Figure 2 illustrates the speedup $S(k)$ for $p_0=0.1$. It is observed that as $k$ increases, $S(k)$ increases and reaches its maximum value at $k=4$, and then decreases. However, when $k$ exceeds 55, $S(k)$ increases again; nevertheless, the increment is very little and not noticeable. Furthermore, the speedup beyond $k=55$ is less than 1, i.e., the pooling method is not effective any more. Therefore, we only need to find the smaller solution of $k$.

Our numerical procedure to find $k$ which satisfies $G(k)=0$ is essentially the standard bisection method (which is described in [3], p. 22), based on the observation is that $G(k)$ is an increasing function of $k$ around the smaller solution of $k$. Since the $k$ found is a real value, we round it to the nearest integers, i.e., the optimal group size is $k^*=\lfloor k \rceil$.

We say that the pooling method is effective, if there is at least one $k\geq 2$, such that $S(k)>1$; and that the pooling method is ineffective, if $S(k)\leq 1$ for all $k\geq 2$. Intuitively, if $p_0$ is too big, the pooling method becomes ineffective. Let $p_0^*$ be the largest value of $p_0$ such that the pooling method is effective. Using numerical verification, we can find that the pooling method is effective when $p_0=0.306$ (with $k=3$ and $S(3)=1.00092$) and ineffective when $p_0=0.307$. Therefore, we can confirm that $p_0^*$ is in the range (0.306,0.307).

### B. Numerical Results

**Fig. 3.**
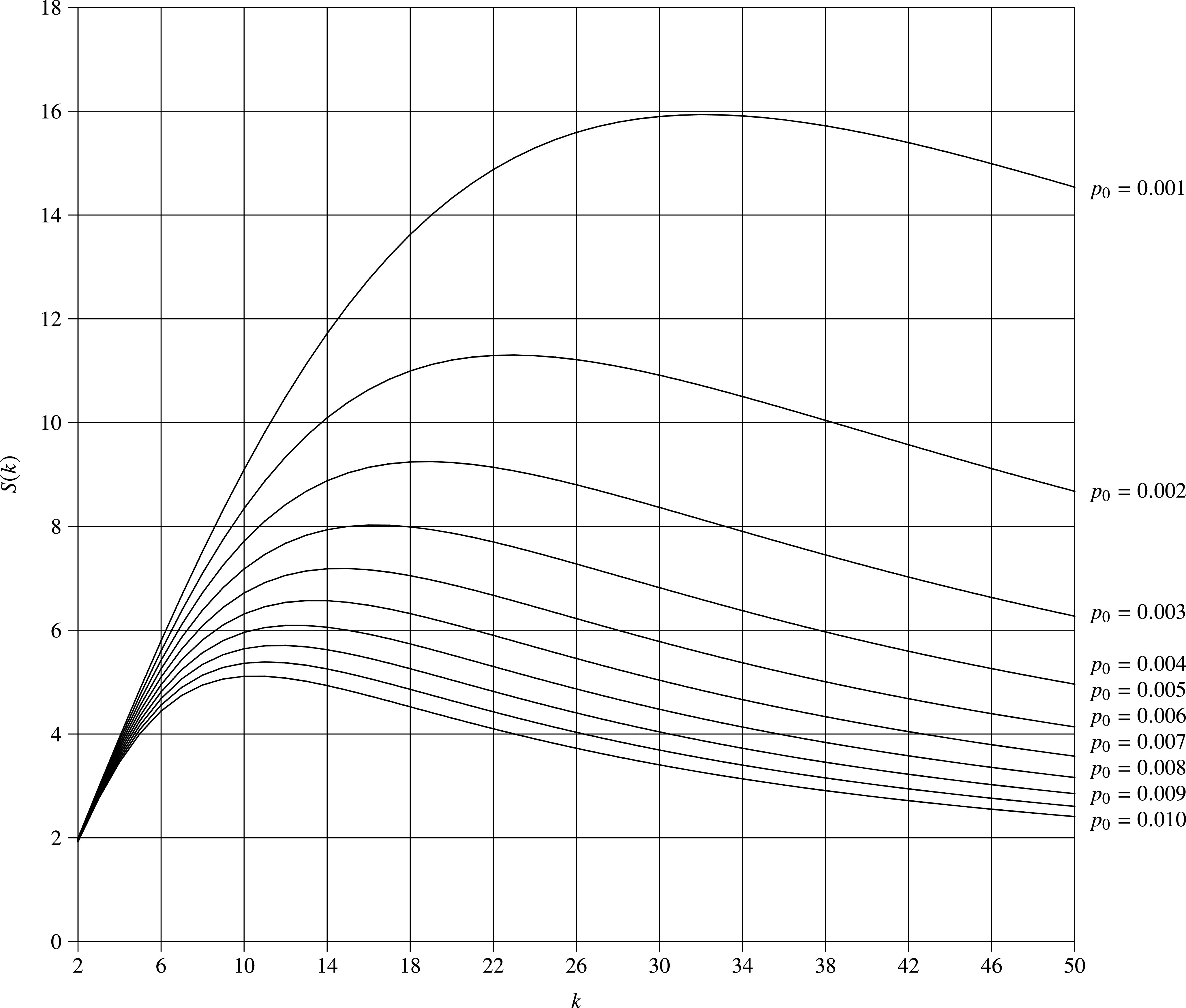
Speedup vs. group size ($p_0=0.001,0.002,0.003,...,0.010$).

In Figure 3, we show the speedup as a function of the group size for $p_0=$ 0.001, 0.002, 0.003, ..., 0.010. It is observed that as $k$ increases, $S(k)$ increases significantly, especially when $p_0$ is small; however, beyond certain point, $S(k)$ decreases noticeably. Hence, there is an optimal choice $k^*$, such that the speedup is maximized.

In Table 1, we demonstrate the optimal group size $k^*$ obtained by our numerical algorithm for $p_0=10^{-1},10^{-2},10^{-3},...,10^{-7}$. We have the following important observations.

• As $p_0$ becomes smaller, the probability $q_1=q_0^{k^*}$ that a group test result is negative becomes higher. For instances, when $p_0=0.01$, the chance for a negative group test result is $q_0^{11}=0.99^{11}=0.8953382$. When $p_0=0.001$, the chance for a negative group test result is $q_0^{32}=0.999^{32}=0.9684911$. Such higher chance will balance the potential higher cost for individual tests in case a group test result is positive.
• As $p_0$ decreases, the optimal group size and the achieved speedup increase rapidly. In particular, we have for $p_0=10^{-r}$,
  \begin{equation*}
k^*>3^r=3^{\log _{10}(1/p_0)}=(1/p_0)^{\log _{10}3}=(1/p_0)^{0.477}.
\end{equation*}
  Furthermore, let $S(p_0)$ be a speedup function of $p_0$. Then we have $S(10^{-(r+1)})/S(10^{-r})>3$, and
  \begin{align*}
&S(10^{-r})>0.56\times 3^r=0.56\times 3^{\log _{10}(1/p_0)}\\
&\quad=0.56(1/p_0)^{\log _{10}3}=0.56(1/p_0)^{0.477}.
\end{align*}
  That is, both $k^*$ and $S(p_0)$ grow sublinearly with $1/p_0$, a quite impressive and nontrivial result.

It is worth to mention that the optimal group size $k^*$ is determined by the fraction $p_0$ of infected people and independent of the size $N$ of the population, since the equation $G(k)=0$ only involves $q_0$ (actually $p_0$), not $N$.

### C. Optimal Group Size

One important (and surprising) observation from Table 1 is that the achieved speed is approximately (and a little bit less than) $k^*/2$, that is, $S(k^*)\approx k^*/2$. Equivalently, for $k^*$, the expected number of tests for one group is approximately 2. This gives us an opportunity to derive a closed-form expression of $k^*$. Let us consider the equation

\begin{equation*}
S(k)={k\over \displaystyle {k+1-kq_0^k}}={k\over \displaystyle {k(1-q_0^k)+1}}={k\over 2},
\end{equation*}

that is,

\begin{equation*}
k(1-q_0^k)=1.
\end{equation*}

Since $G(k)=0$, i.e.,

\begin{equation*}
k={1\over \sqrt{q_0^k\ln (1/q_0)}},
\end{equation*}

we get

\begin{equation*}
1-q_0^k=\sqrt{q_0^k\ln (1/q_0)}.
\end{equation*}

Let $x=\sqrt{q_0^k}$. Then, we have

\begin{equation*}
x^2+\sqrt{\ln (1/q_0)}x-1=0,
\end{equation*}

which gives

\begin{equation*}
x={1\over 2}\left(-\sqrt{\ln (1/q_0)}+\sqrt{\ln (1/q_0)+4}\right),
\end{equation*}

and

\begin{equation*}
q_0^k=x^2=\biggl ({1\over 2}\left(\sqrt{\ln (1/q_0)+4}-\sqrt{\ln (1/q_0)}\right)\biggr)^2,
\end{equation*}

and

\begin{equation*}
k=2\log _{q_0}\biggl ({1\over 2}\left(\sqrt{\ln (1/q_0)+4}-\sqrt{\ln (1/q_0)}\right)\biggr).
\end{equation*}

Since $k^*$ needs to be an integer, we set ($\lfloor z \rceil$ means the nearest integer of $z$)

\begin{equation*}
k^*=\biggl \lfloor 2\log _{q_0}\biggl ({1\over 2}\left(\sqrt{\ln (1/q_0)+4}-\sqrt{\ln (1/q_0)}\right)\biggr)\biggr \rceil +1,
\end{equation*}

which has been verified to be consistent with Table 1.

## III. Intra-Group Acceleration

In this section, we develop our method to find the optimal subgroup size by using *intra-group acceleration*.

### A. The Method

The basic idea of intra-group acceleration is to divide a group into subgroups. When the test result of a group is positive, the group of size $k$ is divided into subgroups of size $m$. For each subgroup, if the test result of the subgroup is negative, only one test is enough; if the test result of the subgroup is positive, $(m+1)$ tests are required, one for subgroup test, and $m$ for individual tests. By using this method, the original $k$ tests for individual samples can possibly be reduced. This method is more effective for small $p_0$ and large $k$.

To develop and analyze intra-group acceleration, we define the following quantities.

• $p_2$: the probability that one subgroup test result is positive under the condition that the test result of a group is positive.
• $q_2$: the probability that one subgroup test result is negative under the condition that the test result of a group is positive.

Note that both $p_2$ and $q_2$ are conditional probabilities. It is clear that $p_2=1-q_2$, and

\begin{equation*}
q_2={q_0^m(1-q_0^{k-m})\over p_1}={q_0^m-q_0^k\over 1-q_0^k},
\end{equation*}

where $p_1$ is the probability that one group test result is positive (i.e., the condition), $q_0^m$ is the probability that all samples in a subgroup are negative (i.e., one subgroup test result is negative), and $(1-q_0^{k-m})$ is the probability that at least one of the remaining $(k-m)$ samples in the same group is positive (to keep the condition).

Under the condition that the test result of a group is positive, the expected number of tests for one subgroup using the pooling method is

\begin{eqnarray*}
T_{\text{subgroup}}(m) &=&1\times q_2+(m+1)\times p_2\\
&=&q_2+(m+1)(1-q_2)\\
&=&m+1-mq_2.
\end{eqnarray*}

Under the condition that the test result of a group is positive, the expected number of tests for one group using intra-group acceleration is

\begin{eqnarray*}
T^{\prime }_{\text{group}} &=&\left({k\over m}\right)T_{\text{subgroup}}(m)\\
&=&\left({k\over m}\right)(m+1-mq_2)\\
&=&k\left(1+{1\over m}-q_2\right)\\
&=&k\biggl (1+{1\over m}-{q_0^m-q_0^k\over 1-q_0^k}\biggr).
\end{eqnarray*}

Since the number of tests for one group without intra-group acceleration is $k$, the speedup of the intra-group acceleration method is

\begin{equation*}
S_{\text{group}}(m) ={k\over T^{\prime }_{\text{group}}} =1\bigg /\biggl (1+{1\over m}-{q_0^m-q_0^k\over 1-q_0^k}\biggr).
\end{equation*}

To maximize $S_{\text{group}}(m)$, we need to minimize

\begin{equation*}
F(m)={1\over m}-{q_0^m-q_0^k\over 1-q_0^k}.
\end{equation*}

It is noticed that

\begin{equation*}
{\partial F(m)\over \partial m}=-{1\over m^2}-{q_0^m\ln q_0\over 1-q_0^k}.
\end{equation*}

To have ${\partial F(m)/\partial m}=0$, we need

\begin{equation*}
{1\over m^2}={q_0^m\over 1-q_0^k}\ln {1\over q_0},
\end{equation*}

which implies that $m$ satisfies

\begin{equation*}
m=\sqrt{\displaystyle {(1-q_0^k)\left({1\over q_0}\right)^m}\over \displaystyle {\ln {1\over q_0}}}.
\end{equation*}

Again, there is no analytical and closed-form solution to the above equation of $m$ at this stage. However, $m$ can be obtained by a numerical algorithm (i.e., the standard bisection method) based on the observation is that

\begin{equation*}
G(m)=m-\sqrt{1-q_0^k\over q_0^m\ln (1/q_0)}
\end{equation*}

is an increasing function of $m$. We use $m^*$ to represent the solution to the equation $G(m)=0$ rounded to the nearest integer.

The expected number of tests for one group using intra-group acceleration is

\begin{equation*}
T_{\text{group}} =1\times q_1+(T^{\prime }_{\text{group}}+1)\times p_1,
\end{equation*}

where $T^{\prime }_{\text{group}}$ can be more accurately expressed as

\begin{equation*}
T^{\prime }_{\text{group}} =\biggl \lfloor {k\over m}\biggr \rfloor T_{\text{subgroup}}(m) +T_{\text{subgroup}}(k\;\text{mod}\;m).
\end{equation*}

The total number of tests for a population of $N$ (which is divided into $N/k$ groups) using both inter-group acceleration and intra-group acceleration is

\begin{equation*}
T_{\text{pooling}} =\left({N\over k}\right)T_{\text{group}}.
\end{equation*}

The speedup of the pooling strategy with both inter-group acceleration and intra-group acceleration is

\begin{equation*}
S(k,m) ={N\over T_{\text{pooling}}} ={k\over T_{\text{group}}}.
\end{equation*}

### B. Numerical Results

In Table 2, we demonstrate the optimal subgroup size $m^*$ (given the optimal group size $k^*$ of Section II) obtained by our numerical algorithm for $p_0=10^{-2},10^{-3},...,10^{-7}$. We have the following important observations.

• It is observed that by using the method of intra-group acceleration, the expected number $T^{\prime }_{\text{group}}$ of tests for one group is noticeably reduced and noticeable speedup $S_{\text{group}}(m^*)$ within a group can be obtained. Of course, such speedup is gained with probability $p_1$, i.e., when the test result of a group is positive.
• The speedup $S(k^*,m^*)$ of the pooling strategy with both inter-group acceleration and intra-group acceleration is noticeably improved. Furthermore, as $p_0$ becomes smaller, the ratio $S(k^*,m^*)/k^*$ increases, which means that $S(k^*,m^*)$ is closer to $k^*$, and the speedup can almost be doubled (compared with Table 1).
• It is worth to mention that when $k^*$ is too small (e.g., $k^*=4$ for $p_0=10^{-1}$), the method of intra-group acceleration is not effective and does not lead to fewer number of tests.

### C. Optimal Subgroup Size

One important observation from Table 2 is that the speedup of the intra-group acceleration method is approximately $m^*/2$, that is, $S_{\text{group}}(m^*)\approx {m^*/2}$. This gives us an opportunity to derive a closed-form expression of $m^*$. Let us consider the equation

\begin{equation*}
S_{\text{group}}(m) =1\bigg /\biggl (1+{1\over m}-{q_0^m-q_0^k\over 1-q_0^k}\biggr) ={m\over 2},
\end{equation*}

that is,

\begin{equation*}
\biggl (1-{q_0^m-q_0^k\over 1-q_0^k}\biggr)m=1,
\end{equation*}

and

\begin{equation*}
\biggl ({1-q_0^m\over 1-q_0^k}\biggr)m=1.
\end{equation*}

Since $G(m)=0$, i.e.,

\begin{equation*}
m=\sqrt{1-q_0^k\over q_0^m\ln (1/q_0)},
\end{equation*}

we get

\begin{equation*}
1-q_0^m=\sqrt{q_0^m(1-q_0^k)\ln (1/q_0)}.
\end{equation*}

Let $x=\sqrt{q_0^m}$. Then, we have

\begin{equation*}
x^2+\sqrt{(1-q_0^k)\ln (1/q_0)}x-1=0,
\end{equation*}

which gives

\begin{equation*}
x={1\over 2}\left(-\sqrt{(1-q_0^k)\ln (1/q_0)}+\sqrt{(1-q_0^k)\ln (1/q_0)+4}\right),
\end{equation*}

and

\begin{eqnarray*}
&&q_0^m=x^2=\biggl ({1\over 2}\biggl (\sqrt{(1-q_0^k)\ln (1/q_0)+4}\\
&&\qquad \qquad \qquad -\sqrt{(1-q_0^k)\ln (1/q_0)}\biggr)\biggr)^2,
\end{eqnarray*}

and

\begin{eqnarray*}
&&m=2\log _{q_0}\biggl ({1\over 2}\biggl (\sqrt{(1-q_0^k)\ln (1/q_0)+4}\\
&&\qquad \qquad \qquad -\sqrt{(1-q_0^k)\ln (1/q_0)}\biggr)\biggr).
\end{eqnarray*}

Since $m^*$ needs to be an integer, we set

\begin{eqnarray*}
&&m^*=\biggl \lfloor 2\log _{q_0}\biggl ({1\over 2}\biggl (\sqrt{(1-q_0^k)\ln (1/q_0)+4}\\
&&\qquad \qquad \qquad -\sqrt{(1-q_0^k)\ln (1/q_0)}\biggr)\biggr)\biggr \rceil,
\end{eqnarray*}

which has been verified to be consistent with Table 2.

## IV. Joint Optimization

Our optimal group size $k^*$ in Section II is obtained based on the assumption that the method of intra-group acceleration is not used. With the reduced number $T^{\prime }_{\text{group}}$ of tests for one group by using intra-group acceleration, it is likely that $k^*$ can be increased, which creates more room for improving the speedup. The increased group size certainly affects the choice of the optimal subgroup size $m^*$. Fortunately, based on the analytical expressions of $m^*$ in Section III, it is possible to conduct *joint optimization* for both inter-group acceleration and intra-group acceleration, i.e., to simultaneously find the optimal group size and the optimal subgroup size, when both inter-group acceleration and intra-group acceleration are involved.

### A. The Method

Recall that

\begin{eqnarray*}
&&m(k)={2\over \ln {q_0}}\ln \biggl ({1\over 2}\biggl (\sqrt{(1-q_0^k)\ln (1/q_0)+4}\\
&&\qquad \qquad \qquad -\sqrt{(1-q_0^k)\ln (1/q_0)}\biggr)\biggr),
\end{eqnarray*}

and

\begin{equation*}
T^{\prime }_{\text{group}}(k) =k\biggl ({1\over m(k)}+{1-q_0^{m(k)}\over 1-q_0^k}\biggr),
\end{equation*}

and

\begin{equation*}
T_{\text{group}}(k) =q_0^k+(T^{\prime }_{\text{group}}(k)+1)(1-q_0^k),
\end{equation*}

and

\begin{equation*}
S(k,m(k))={k\over T_{\text{group}}(k)},
\end{equation*}

where $S(k,m(k))$, as well as $m(k)$, $T^{\prime }_{\text{group}}(k)$, and $T_{\text{group}}(k)$ are all viewed as functions of $k$.

**Fig. 4.**
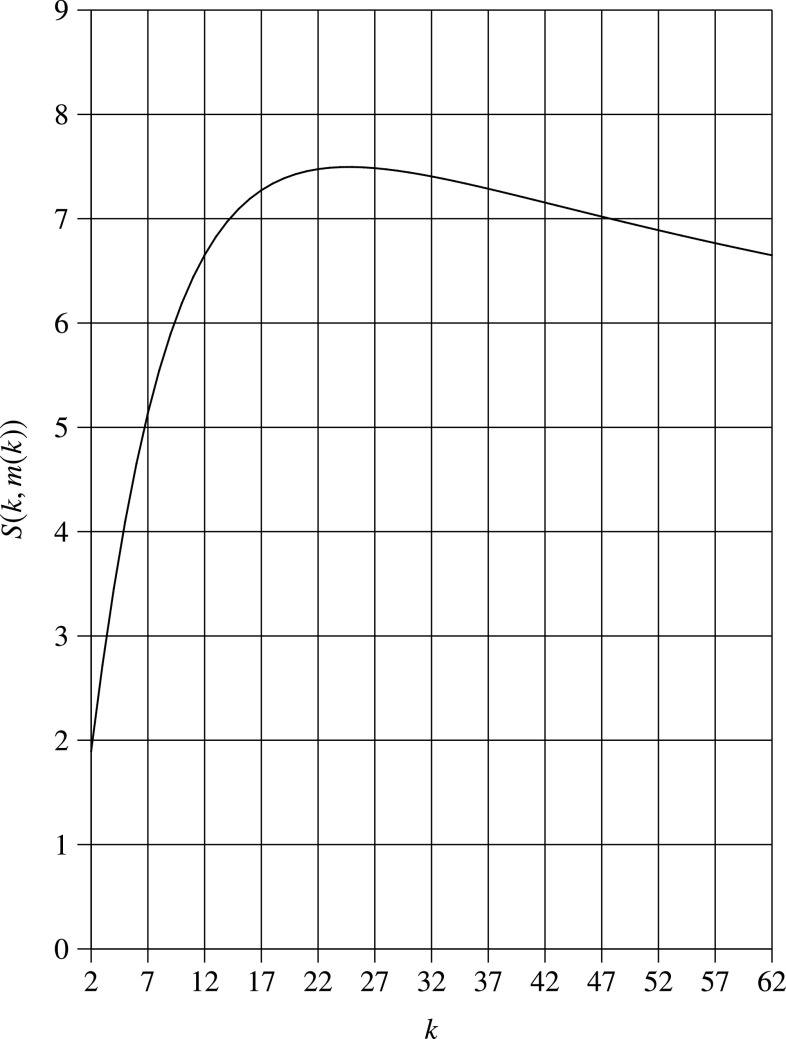
Speedup $S(k,m(k))$ vs. group size $k$ ($p_0=0.01$).

In Figure 4, we display the speedup $S(k,m(k))$ for $p_0=0.01$. It is clear that $S(k,m(k))$ is a concave function of $k$, and there is an optimal choice of $k=25$, which maximizes $S(k,m(k))$.

To maximize $S(k,m(k))$, we need ${\partial S(k,m(k))/\partial k}=0$, where

\begin{equation*}
{\partial S(k,m(k))\over \partial k}= {1\over T_{\text{group}}(k)} -{k\over T^2_{\text{group}}(k)}\cdot {\partial T_{\text{group}}(k)\over \partial k},
\end{equation*}

and

\begin{eqnarray*}
{\partial T_{\text{group}}(k)\over \partial k} &=&q_0^k\ln {q_0} +{\partial T^{\prime }_{\text{group}}(k)\over \partial k}(1-q_0^k)\\
&&\qquad -(T^{\prime }_{\text{group}}(k)+1)q_0^k\ln {q_0}\\
&=&{\partial T^{\prime }_{\text{group}}(k)\over \partial k}(1-q_0^k) -T^{\prime }_{\text{group}}(k)q_0^k\ln {q_0},
\end{eqnarray*}

and

\begin{eqnarray*}
&&{\partial T^{\prime }_{\text{group}}(k)\over \partial k} =\biggl ({1\over m(k)}+{1-q_0^{m(k)}\over 1-q_0^k}\biggr)\\
&&\qquad \qquad \qquad +k\biggl (-\biggl ({1\over m^2(k)}+{q_0^{m(k)}\ln {q_0}\over 1-q_0^k}\biggr){\partial m(k)\over \partial k}\\
&&\qquad \qquad \qquad \qquad +{(1-q_0^{m(k)})q_0^k\ln {q_0}\over (1-q_0^k)^2}\biggr),
\end{eqnarray*}

and

\begin{align*}
&{\partial m(k)\over \partial k} ={1\over \sqrt{(1-q_0^k)\ln (1/q_0)+4}-\sqrt{(1-q_0^k)\ln (1/q_0)}}\\
&\quad \times \left({1\over \sqrt{(1-q_0^k)\ln (1/q_0)}} - {1\over \sqrt{(1-q_0^k)\ln (1/q_0)+4}} \right)\\
&\quad\times q_0^k\ln (1/q_0).
\end{align*}

The equation ${\partial S(k,m(k))/\partial k}=0$ can be solved by using the standard bisection method, based on the observation is that ${\partial S(k,m(k))/\partial k}$ is a decreasing function of $k$.

Once the optimal group size $k^*$ is available, the corresponding optimal subgroup size $m(k^*)$ and the speedup $S(k^*,m(k^*))$ can be calculated easily.

### B. Numerical Results

In Table 3, we demonstrate the optimal group size $k^*$, the optimal subgroup size $m(k^*)$, and the speedup $S(k^*,m(k^*))$ for $p_0=10^{-1},10^{-2},10^{-3},...,10^{-7}$. We have the following important observations.

• The optimal group size $k^*$ is significantly greater than the optimal group size of inter-group acceleration (see Table 1). In particular, we have for $p_0=10^{-r}$,
  \begin{eqnarray*}
&&k^*\geq (25/16)4^r=1.5625\times 4^{\log _{10}(1/p_0)}\\
&&=1.5625(1/p_0)^{\log _{10}4}=1.5625(1/p_0)^{0.602}.
\end{eqnarray*}
  That is, the optimal group size grows sublinearly with $1/p_0$.
• The optimal subgroup size $m(k^*)$ is noticeably greater than the optimal subgroup size of intra-group acceleration (see Table 2). In particular, we have for $p_0=10^{-r}$,
  \begin{equation*}
m^*\geq 2^r=2^{\log _{10}(1/p_0)}=(1/p_0)^{\log _{10}2}=(1/p_0)^{0.301}.
\end{equation*}
  That is, the optimal subgroup size grows sublinearly with $1/p_0$.
• The achieved speedup $S(k^*,m(k^*))$ with joint optimization for both inter-group acceleration and intra-group acceleration is significantly greater than the speedup of intra-group acceleration with given and fixed group sizes (see Table 2). The most important factor that determines the gap is the optimality of $k^*$. Furthermore, if the speedup is regarded as a function $S(p_0)$ of $p_0$, as $r$ increases, we have $S(10^{-(r+1)})/S(10^{-r})>4$, and for $p_0=10^{-r}$, we have
  \begin{eqnarray*}
&&S(10^{-r})>(11/16)4^r=0.6875\times 4^{\log _{10}(1/p_0)}\\
&&=0.6875(1/p_0)^{\log _{10}4}=0.6875(1/p_0)^{0.602}.
\end{eqnarray*}
  That is, the speedup grows sublinearly with $1/p_0$. Also notice that $S(k^*,m(k^*))$ is approximately (and a little bit less than) $k^*/2$ for small $p_0$.
• It is worth to mention that such two-level joint optimization is effective and applicable to any $p_0$.

## V. Practical Issues

We would like to mention the following issues related to the applicability of the research in this paper.

• *Availability of $p_0$* – In this paper, it has been assumed that the value of $p_0$, i.e., the fraction of infected people, is available in advance. In reality, the value of $p_0$ can be estimated accurately by testing a group of $n$ random samples, where $n$ is reasonably large but still much less than the population size $N$, so that the time spent to obtain $p_0$ is negligible and does not reduce the speedup too much.
• *Independence of Samples* – In this paper, it has been assumed that individual sample test results are independent of each other. In reality, there might be correlation among individual sample test results. Such correlation exists for people from the same family, the same company, the same school, and so on. One effective way to reduce the impact of sample correlation is to randomize the samples, so that people from the same social group are not tested together. Theoretically, the impact of such dependency on our methods, analysis, and algorithms needs deeper investigation.
• *Limitation on Group Size* – In this paper, it has been assumed that the group size and the subgroup size can be arbitrarily large. In practice, there can be limitation on the number of samples that can be combined into one test. The impact of such restriction on the performance of our hierarchical pooling strategy deserves more careful study.
• *Applications in Real Testing* – Although we believe that our hierarchical pooling strategy can be readily applied to accelerating asymptomatic COVID-19 screening of any scale, it is still exciting to actually use our methodology in a real community, city, or country. However, such effort which involves joint endeavor of social, medical, and governmental agencies, is certainly beyond the scope of this paper.
• *Generality of Our Strategy* – Although our hierarchical pooling strategy has been developed for asymptomatic COVID-19 testing, we believe that our general-purpose analytical methods and numerical algorithms are also applicable to accelerating the testing of other deceases that have already been existing or may appear in the future.

## VI. Conclusion

We have developed a two-level hierarchical pooling strategy for accelerating asymptomatic COVID-19 screening. We have also been able to determine the optimal group size and the optimal subgroup size, which minimize the total number of tests, maximize the speedup of the hierarchical pooling strategy, and minimize both time and cost of testing. It is found that the optimal group size, the optimal subgroup size, and the achieved speedup grow sublinearly with the reciprocal of the fraction of infected people. Our method is effective in supporting faster and cheaper asymptomatic COVID-19 screening.

There are further research directions. One challenge is to derive a closed-form expression of the optimal group size for our two-level hierarchical pooling strategy. For another further investigation, we notice that the hierarchical testing system in this paper has only two levels, i.e., group and subgroup. It is interesting to consider a hierarchical acceleration system with more levels (e.g., for very small $p_0$ and not too small $m(k^*)$), in which, there are groups, which are divided into subgroups, which are further divided into sub-subgroups, and so on, with group level, subgroup level, and sub-subgroup level acceleration. For such a multi-level testing system, it is necessary to determine the optimal group, subgroup, and sub-subgroup sizes. It is conceivable that such a *multi-level acceleration mechanism* is more powerful and more effective in producing higher speedup. We believe that the analytical approach and algorithmic procedure developed in this paper can be extended towards this direction.

**TABLE 1:** Optimal Group Size ($p_0=10^{-1},10^{-2},10^{-3},...,10^{-7}$)

$p_0$	$k^*$	$q_1$	$S(k^*)$	$S(k^*)/k^*$
$10^{ -1}$	4	0.65610	1.68379	0.42095
$10^{ -2}$	11	0.89534	5.11324	0.46484
$10^{ -3}$	32	0.96849	15.93399	0.49794
$10^{ -4}$	101	0.98995	50.12366	0.49627
$10^{ -5}$	317	0.99684	158.23859	0.49918
$10^{ -6}$	1001	0.99900	500.12486	0.49963
$10^{ -7}$	3163	0.99968	1581.26380	0.49993

**TABLE 2:** Optimal Subgroup Size ($p_0=10^{-2},10^{-3},...,10^{-7}$)

$p_0$	$k^*$	$m^*$	$S_{\text{group}}(m^*)$	$S_{\text{group}}(m^*)/m^*$	$S(k^*,m^*)$	$S(k^*,m^*)/k^*$
$10^{ -2}$	11	3	1.58632	0.52877	6.37402	0.57946
$10^{ -3}$	32	6	2.70606	0.45101	23.31338	0.72854
$10^{ -4}$	101	10	4.82190	0.48219	83.43650	0.82610
$10^{ -5}$	317	18	8.85879	0.49215	284.75052	0.89827
$10^{ -6}$	1001	32	15.68754	0.49024	940.93053	0.93999
$10^{ -7}$	3163	56	28.05045	0.50090	3054.08904	0.96557

**TABLE 3:** Optimal Group and Subgroup Sizes ($p_0=10^{-1},10^{-2},10^{-3},...,10^{-7}$)

$p_0$	$k^*$	$m(k^*)$	$S(k^*,m(k^*))$	$S(k^*,m(k^*))/k^*$
$10^{ -1}$	8	2	3.17460	0.39683
$10^{ -2}$	25	5	11.23470	0.44939
$10^{ -3}$	106	10	51.57542	0.48656
$10^{ -4}$	476	22	232.63757	0.48873
$10^{ -5}$	2179	46	1088.34934	0.49947
$10^{ -6}$	10051	100	5012.84195	0.49874
$10^{ -7}$	46525	215	23259.28096	0.49993
